# Early changes in tear film protein profiles after femtosecond LASIK surgery

**DOI:** 10.1186/s12014-020-09303-9

**Published:** 2020-10-19

**Authors:** Janika Nättinen, Petri Mäkinen, Ulla Aapola, Lasse Orsila, Juhani Pietilä, Hannu Uusitalo

**Affiliations:** 1 SILK, Department of Ophthalmology, Faculty of Medicine and Health Technology, Tampere University, PL 100, 33014 Tampere, Finland; 2 Silmäasema Eye Hospital, Tampere, Finland; 3 TAUH Eye Center, Tampere University Hospital, Tampere, Finland

**Keywords:** Refractive surgery, Femtosecond laser, LASIK, Tear proteomics, Wound healing

## Abstract

**Background:**

Femtosecond laser-assisted in situ keratomileusis (LASIK) has proven to be an efficacious, predictable, and safe procedure for the correction of refractive errors. We examined the early tear protein changes of patients undergoing LASIK surgery in order to better understand the mechanisms and proteins related to laser corneal surgery and initial recovery.

**Methods:**

Corneal flaps were created with Ziemer FEMTO LDV Z6 I femtosecond laser and stroma was ablated using Wavelight EX500 excimer laser. Tear samples were collected preoperatively as well as 1.5 h and 1 month after LASIK treatment using glass microcapillary tubes. Relative quantification of tear proteins was performed with sequential window acquisition of all theoretical fragment ion spectra mass spectrometry (SWATH-MS).

**Results:**

SWATH-MS revealed that 158 proteins had altered expression levels 1.5 h after the operation. Two-thirds of these proteins, mostly connected to migration and inflammation response, returned to preoperative levels within the first postoperative month. The other proteins, which did not return to baseline levels, included proteins connected to for example epithelial barrier function. We also identified several proteins, which correlated with surgical variables, such as the amount of correction, flap thickness and flap diameter.

**Conclusions:**

The present study showed that an uneventful femtosecond LASIK refractive surgery induced a significant immune cell migration and inflammation-associated changes in tear proteomics profile quickly after the operation, but the expression of most proteins recovered almost completely to the preoperative levels within the first month. The individual proteins identified in our study are potential targets for the follow-up and modification of LASIK-induced biochemical processes.

## Background

Laser-assisted in situ keratomileusis (LASIK) is a safe and effective procedure for the correction of refractive errors. Therefore, it is one of the most frequently used laser eye surgery procedures and the patient satisfaction is generally high [1]. The operation is performed by creating a thin flap in the epithelium of cornea, for example with femtosecond laser, and then reshaping the underlying corneal layer (stroma). Despite the very good clinical results, there are variations in LASIK’s effects and complications, which indicate individual differences in corneal and ocular surface response to surgical trauma and wound healing. Increased dry eye symptoms, reduced tear film stability and reduced corneal sensation are common after LASIK surgery, possibly due to the corneal nerve disruption and related changes on the ocular surface caused by the surgery [2–4]. However, these post-LASIK side effects are usually temporary and disappear during the first months, although a small subset of patients can develop a long term, chronic condition [5]. The depth of laser treatment, higher refractive correction, flap thickness, hinge parameters and sex have been identified to influence the likelihood of these side effects [2, 6, 7].

It is known that corneal refractive surgical procedures initiate a complex cascade of healing responses in cornea and ocular surface. Corneal trauma triggers an immediate release of various cytokines and growth factors, which induce interactions between epithelial cells, keratocytes, corneal nerves, lacrimal glands, tear film, and cells of the immune system [8–11]. The tear fluid, which reflects the state of the underlying eye tissue, is not only an important part and mediator of the wound healing process, but it is also a non-invasive sampling material, making it an ideal analysis target to study the effects of ocular surgery. Therefore, we decided to analyse the early tear protein changes of patients undergoing LASIK surgery in order to better understand the mechanisms and proteins related to laser corneal surgery and initial recovery. Previously, tear proteomics evaluating the changes caused by LASIK surgery have been studied using multiplex immunoassays [12, 13], isobaric tags for relative and absolute quantitation (iTRAQ) [14] and enzyme-linked immunosorbent assay (ELISA) [15, 16]. Our aim was to study the immediate protein changes caused by surgery and to identify proteins related to normal, early recovery processes after surgery by implementing the sequential window acquisition of all theoretical mass spectra (SWATH-MS) method, which enables the identification and quantification of hundreds of proteins from each individual tear sample.

## Methods

### Study design and patients

Seventy patients were evaluated preoperatively as well as postoperatively at 1.5 h and, on average, at 1 month after the surgery. Altogether 70 patients were included in the study and both eyes were initially sampled for tears. Exclusion criteria included previous eye surgery, glaucoma, dry eye symptoms and pregnancy. In proteomics analyses comparing previous visits against 1 month follow-up visit, only patients with a follow-up visit within 30 ± 15 days from the operation were included.

### Surgery protocol and clinical tests

#### Preoperative examinations

Before the femtosecond LASIK surgery, all patients underwent a complete preoperative ophthalmologic examination, which was performed in order to identify any severe pathology that might be a contraindication for surgery. The preoperative examination included biomicroscopy, determination of refraction, wavefront analysis (Allegro Analyzer, Wavelight AG, Erlangen, Germany) and measurements of three-dimensional corneal topography (Allegro Oculyzer, Wavelight AG, Erlangen, Germany), uncorrected and corrected distance visual acuity (UDVA and CDVA, respectively), and intraocular pressure (Nidek Tonoref RKT-7700, Gamagori, Aichi, Japan).

#### Surgical procedure

Prior to the surgery, various drops were instilled into the eyes. These included antibiotic levofloxacin 5 mg/ml eye drops (Oftaquix, Santen Oy, Tampere, Finland), diclofenac 1 mg/ml (Voltaren Ophtha, THEA, Clermont-Ferrand, France) for pain and inflammation, brimonidine tartrate 2 mg/ml (Alphagan, Allergan, Westport, Ireland) for conjunctival vessel construction and oxybuprocaine hydrochloride 4 mg/ml (Oftan Obucain, Santen Oy) as a topical anesthetic. Topical medication is an essential element in refractive surgery and therefore, tear fluid changes cannot be studied without medication. An aspirating speculum (Geuder, no 15961, Heidelberg, Germany) was used to keep the eyelid open. Preoperative corneal thickness was measured with an ultrasonic pachymetry (SP-3000, Tomey Corp., Nagoya, Japan).

Femtosecond laser FEMTO LDV Z6 I (Ziemer Ophthalmic Systems, Port, Switzerland), which delivered 100 nJ pulse energy and > 2 MHz repetition rate, was used for the flap creation. The target flap thickness ranged from 90 to 110 µm and all flaps were roundly shaped and set at a 60-90º angled edge. Plastic single-use suction rings with a 9.5 mm diameter were used with a target flap diameter of 9.3 mm. The target hinge length was 4.0 mm in all cases. Vacuum pressure was 700 mbar and cutting time 28 s.

The excimer laser treatment was done on the exposed stroma using the Wavelight EX500 excimer laser (Wavelight AG, Erlangen, Germany). The optical zone ranged from 6.5 to 7.0 mm and the treatment zone was less than 9.0 mm. All complications during the procedure and follow-up time were recorded.

#### Postoperative treatment

Thirty minutes after the LASIK operation, moisture drops sodium hyaluronate 0.15 mg/ml (Oxyal, Dr Gerhard Mann Chem. –pharm. Fabrik GmbH, Berlin, Germany) were instilled into the eyes. Topical anaesthetic oxybuprocaine hydrochloride (Oftan Obucain) was instilled into the eyes one hour after the surgery. Dexamethasone and chloramphenicol containing drops (Oftan Dexa-Chlora, Santen Oy) with the tapered dose were used for the first week, starting 3 h after the surgery.

### Tear fluid collection

**Fig. 1:**
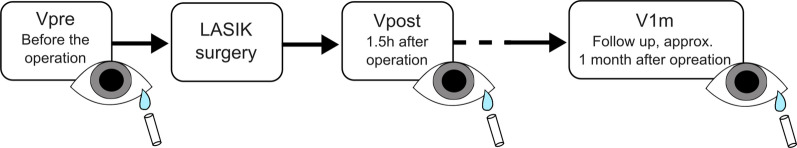
Study outline. The initial capillary sample was taken before the operation (Vpre) and the postoperative sample (Vpost) was taken 1.5 h after the LASIK surgery. The final follow-up visit and sampling took place approximately 1 month after the surgery (V1m)

Patients were asked to participate in a tear fluid collection before LASIK treatment (Vpre) and postoperatively 1.5 h after LASIK (Vpost), and at follow-up approximately 1 month after LASIK (V1m) (Fig. 1). The Vpre tear sample was taken before the installation of any eye drops. Tear samples were collected into 2 or 3 µl glass microcapillary tubes and stored first at −20ºC and then transferred to −80ºC until assessed.

### Tear sample preparation

A detailed description of the sample preparation steps has been previously described [17, 18]. In brief, samples were flushed from capillaries with 0.5% sodium dodecyl sulphate (SDS) and protein concentration was measured with DC protein assay kit (Bio-Rad laboratories Inc, Hercules, USA). 15/210 samples did not have sufficient amounts of proteins (≥ 5 µg) for further analysis. Acetone-precipitated proteins were dissolved in 2% SDS, reduced by tris-(2-carboxyethyl)phosphine (TCEP) and alkylated with iodoacetamide (IAA) on 30 kDa molecular weight cut-off filters (Pall Corporation, Port Washington, NY, USA) in order to lower the sample complexity and remove small contaminants. Samples were digested with trypsin (Sciex, Framingham, USA) and cleaned and desalted with C18 tips (Thermo Fisher Scientific). Unless otherwise stated all reagents were purchased from Sigma-Aldrich (St. Louis, MO, USA).

### Identification and SWATH-MS quantification of tear proteins

The samples were analysed using the Eksigent 425 NanoLC coupled to the high speed TripleTOF™ 5600 + mass spectrometer (Ab Sciex, Concord, Canada). Equal amounts (3 µg) of tear peptide mix from each sample were first loaded into the trap column (ChromXP C18-CL 3 µm 120 Å, 200 µm × 0.5 mm) and then separated using a cHiPLC column (ChromXP C18-CL 3 µm 120 Å, 75 µm × 15 cm). Peptides were introduced into the mass spectrometer (MS) via NanoSpray III source and analysed with 120 min 6 step gradients using eluent A (0.1% FA in 1% ACN) and eluent B (0.1% FA in ACN) at 300 nl/min. The spectral library for the SWATH analysis was created by the data dependent-acquisition (DDA) method using the ProteinPilot® software version 4.7 (Ab Sciex, Concord, Canada) and false discovery rate (FDR) < 1%. For protein identification, MS/MS spectra were searched against the UniProtKB/Swiss-Prot human database (version 2016_01). Protein quantification against the obtained spectral library was performed using PeakView® and MarkerView® software (Sciex, Redwood City, USA). Equal amounts of proteins were used, and two replicate MS-analyses were performed from each sample. Due to instrument errors (column or trap column break down) and small sample size (not enough sample for additional injections) 3 samples were lost. Ten peptides from albumin were chosen for the retention time calibration for all samples. Five transitions per peptide and 1–15 peptides were used for the peak area calculations. The most relevant/significant differentially expressed proteins were subjected to manual inspection of peptides, including correct peak selection check (FDR 1%, 99% peptide confidence level) and chromatogram inspection in relation to library chromatogram. Protein quantification results are presented as a combination of protein specific peptides peak intensities from SWATH-MS measurement and here referred to as protein expression. The key parameters used in MS analysis and protein identification have been described in detail before [17, 18].

### Statistical analysis

Only one eye from each patient was included in the analysis of the proteomics data. The quantified proteomics data was log_2_-transformed and the quantile normalization was applied to reduce any potential bias. The intraclass correlation coefficient (ICC) was used to establish the quality of the MS analysis replicates. The means were taken from the MS analysis replicates. The differential expression analyses between visits, i.e. preoperative (Vpre), postoperative (Vpost) and one average one month after the operation (V1m), were performed using the Wilcoxon signed rank test with the continuity correction to account for the repeated measures.

The Spearman’s rank correlation was implemented to measure correlation between continuous clinical variables (flap thickness and correction (target refraction—preoperative spherical equivalent refraction (SEQ))) and protein expression changes between visits. The correlation coefficients were estimated for myopic patients only, due to differences in surgical strategies and a low number of hyperopic patients. In addition, protein expression level differences between sexes, flap diameter (< 9.4 mm vs ≥ 9.4 mm) and hinge length (< 4 mm vs ≥ 4 mm) were evaluated with Wilcoxon rank sum test. Flap diameter and hinge length variables were discretized into two groups since both variables were restricted to a small number of possible values.

The multiple testing was accounted for by implementing the Benjamini–Hochberg adjustment to the raw p-values. A significance level (alpha) of 0.05 was used as a threshold in all tests, excluding the Spearman’s correlation, where a threshold on absolute correlation coefficient (Rho) of 0.5 was implemented. The R software version 3.2.3 [19] was used to perform the analyses. Online database tool STRING [20] was used to identify protein–protein interaction networks (the minimum required interaction score set as “high confidence”) and the IPA (Ingenuity Pathway Analysis) software (IPA, QIAGEN Redwood City, USA) was used to identify enriched biological functions.

## Results

### Patient demography

In total, 70 patients were included in the study. The mean age of the patients was 38.4 ± 10.3 years and ranged from 18 to 57 years. The data consisted of 42 females and 28 males and the mean ages of the two groups did not differ significantly (*P* = 0.79). Refractive corrections were performed for both myopic and hyperopic subjects (59 and 11 patients, respectively) and hence, these two groups are shown separately in Table 1, which summarizes further the clinical information.

**Table 1:** Clinical information of the patients

Clinical variable	Myopic (n = 59)		Hyperopic (n = 11)	
	Vpre	V1m	Vpre	V1m
SEQ (D)	− 3.9 ± 2	− 0.2 ± 0.7	2 ± 1.1	− 1 ± 0.9
Corneal thickness (µm)	540.9 ± 33.8	479.2 ± 44.4	538 ± 25.9	530.5 ± 24.6
IOP (mmHg)	16.5 ± 2.8	11.7 ± 1.9	16.3 ± 2.7	13.8 ± 3.2
Correction (D)	3.5 ± 1.9		3 ± 1.1	
Flap diameter (mm)	9.3 ± 0.1		9.4 ± 0.1	
Flap thickness (μm)	93.8 ± 6.9		89.5 ± 5	
Predictability (D)	0.2 ± 0.3		0.1 ± 0.4	

The values are shown as mean ± standard deviation

*SEQ*  spherical equivalent refraction, *IOP* intraocular pressure, *Correction* abs(target refraction-preoperative SEQ), *Predictability* abs(postoperative SEQ – target refraction), *Vpre* preoperative visit, *Vpost* postoperative visit 1.5 h after surgery, *V1m* follow-up visit 1 month after surgery

### Proteomics data and differential expression analysis

With a spectral library of 1329 proteins, 747 proteins had distinct peptides and were quantified in all samples. The SWATH-MS was able to produce reproducible results with a mean ICC of 0.97 between MS analysis replicates. Number of peptides used in the quantitation of proteins is listed in Additional file 1.

**Fig. 2:**
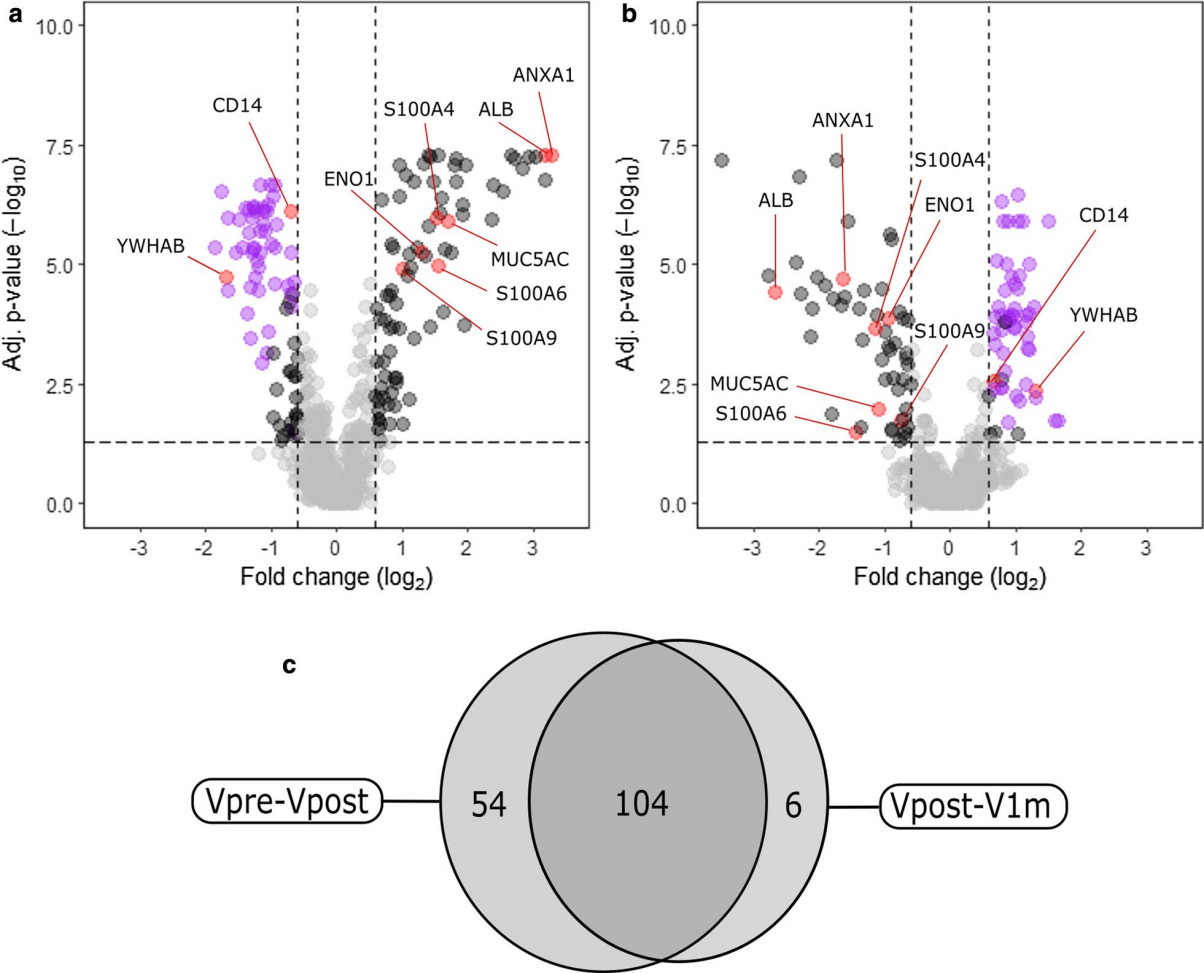
Differentially expressed proteins between pre- and postoperative visits’ tear samples. **a**, **b** The volcano plots display the results of differential expression analyses for **a** Vpre-Vpost (55 sample pairs available) and **b** Vpost-V1m (35 sample pairs available) comparisons. The adjusted p-values (y-axis) are shown in –log_10_ scale and the dashed horizontal line represents the adjusted p-value threshold of 0.05 and the points above this horizontal line are statistically significant. The median fold changes are in the x-axis and are in log_2_ scale. The two dashed lines here are showing the 1.5 and 0.67-fold change thresholds. The values on the left side of the thresholds represent proteins, which were downregulated and the points on the far right were upregulated. Some of the proteins of interest are named (red) and the purple dots represent immunoglobulin subunits. **c** There were altogether 158 significantly differentiated proteins in Vpre-Vpost and 110 in Vpost-V1m comparisons. The results from Vpre-Vpost and Vpost-V1m comparisons share altogether 104 proteins, which are differentially expressed in both comparisons

The comparison of protein expression levels between Vpre and Vpost time points identified altogether 158 differentially expressed proteins (median fold change (FC) > 1.5 or < 0.67, adjusted *p* value < 0.05). Out of these proteins, 81 were upregulated and 77 were downregulated. Most notably, the downregulated protein list included 49 immunoglobulin subunits. These are colored in Fig. 2a, where some other individual proteins are also labeled. The full results can be found from the supplementary materials (Additional file 2).

A similar differential expression comparison was performed for Vpost and V1m time points, and altogether 110 statistically significant proteins were identified to have median FC > 1.5 or < 0.67 and adjusted *p* value < 0.05. Fifty-six proteins were upregulated and 54 were downregulated (Fig. 2b) and full results are listed in Additional file 3. No statistically significant differences were present in comparisons between Vpre and V1m time points.

**Fig. 3:**
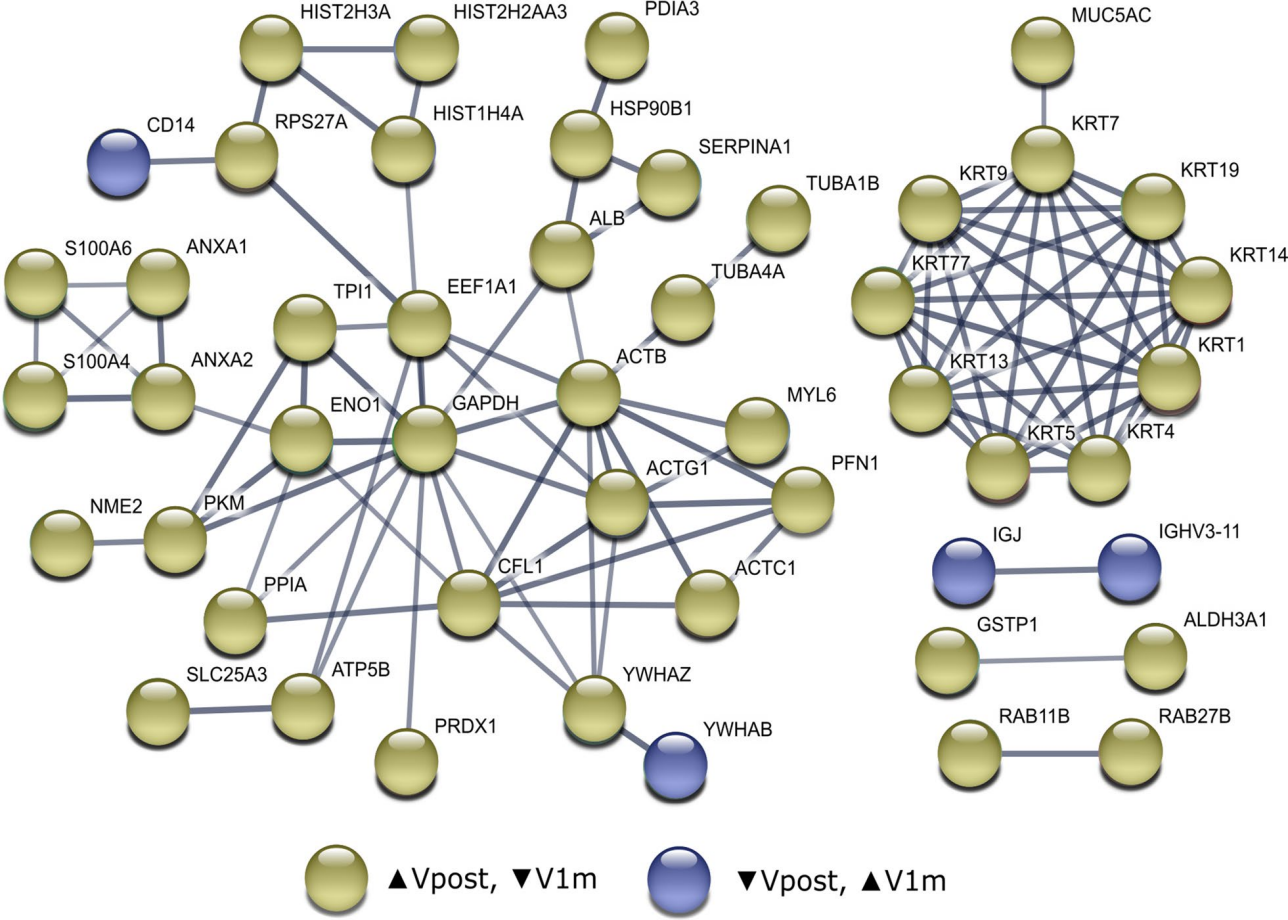
Proteins differing significantly in both time point comparisons (Vpre-Vpost and Vpost-V1m). The protein–protein interaction network displays proteins, which had increased expression levels 1.5 h after the surgery and decreased expression levels 1 month after the surgery (in yellow) and proteins, which were initially decreased 1.5 h post-surgery and increased 1 month after surgery (in blue). Only proteins, which could be identified in STRING and had protein–protein connections are included

As shown in Fig. 2c, there were several proteins, which had changed expression levels in both time points (104 proteins) and it is worth noting, that all these proteins had the opposite direction between the visit comparisons, i.e., the protein expression level changes returned back to preoperative values 1 month after surgery thus indicating recovery after the surgical trauma. This group of proteins included, e.g., albumin (ALB), annexins A1 and A2 (ANXA1, ANXA2), alpha-enolase (ENO1), 14-3-3 protein zeta/delta (YWHAZ) and several S100A proteins (S100A4, S100A6, S100A9), which were initially increased 1.5 h after the surgery, and were then decreased by the final visit 1 month after surgery. Some proteins, such as 14-3-3 protein beta/alpha (YWHAB) as well as 47 out of the 49 immunoglobulin subunits were downregulated 1.5 h post-surgery and returned to normal within 1 month. Figure 3 displays the protein–protein interactions of these common proteins, found from both comparisons.

**Fig. 4:**
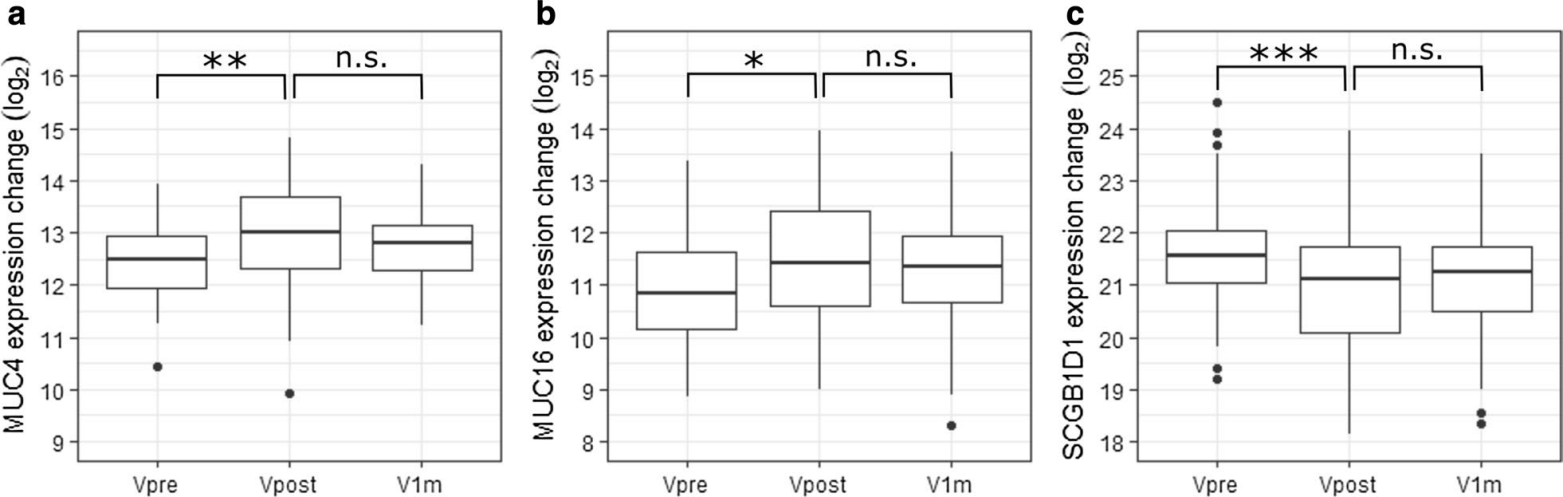
Mucins (MUC4 and MUC16) and secretoglobin family 1D member 1 (SCGB1D1) had significant expression level changes only between Vpre and Vpost time points. **a**, **b** Mucins MUC4 and MUC16 had increased expression levels 1.5 h after the surgery (Vpre-Vpost) and these levels did not reduce between the postoperative visits (Vpost-V1m). **c** Notably decreased expression levels of SCGB1D1 were observed between Vpre and Vpost visits, while no difference was again observed between Vpost and V1m. *P < 0.05; **P < 0.01; ***P < 0.001; *n.s.* non-significant

In addition to these proteins, some proteins were significantly changed only between the Vpre and Vpost visits (54) or had altered expression only 1 month after the surgery (6), as shown in Fig. 2c. The most notably changed proteins, i.e. those with significant changes according to one time point comparison (FC > 1.5 or < 0.67 and *p* value < 0.05) and no notable change in the other time point comparison (FC < 1.25 or > 0.8 and *p* value > 0.05), included some well-known tear fluid proteins visualized in Fig. 4.

### Pathway analysis of the differentially expressed proteins

The pathway analysis was conducted using the IPA and the results obtained from the separate Vpre-Vpost and the Vpost-V1m comparisons are summarized in Table 2. From the enrichment results relating to diseases and biological functions, it is evident that 1.5 h after the surgery there was a rapid activation of pathways related to cell movement of immune cells, e.g. myeloid cells, phagocytes and leukocytes, inflammation, angiogenesis and viral infection. In addition, the pathways related to organismal survival were decreased 1.5 h after the surgery. These actions were then reversed by the final visit, 1 month after the surgery, where the cell movement and viral infection were decreased, and organismal survival and cell death were increased. An increase in vasculature development and energy production only take place when comparing the Vpre and Vpost time points.

**Table 2:** The pathways associated with surgery effects and recovery

Categories	Diseases or Functions Annotation	Vpre vs Vpost			Vpost vs V1m		
		Z-score	Adj *P*	Protein count	Z-score	Adj *P*	Protein count
Organismal survival	Morbidity or mortality	− 4.3	0.007	41	3.5	0.021	29
	Organismal death	− 4.2	0.009	40	3.3	0.028	28
Cell death and survival	Apoptosis				2.1	0.003	34
Protein synthesis	Quantity of enzyme	− 2.2	0.025	5			
Tissue morphology	Quantity of leukocytes	− 2.4	0.005	21			
	Quantity of blood cells	− 2.1	0.007	22			
Inflammatory response	Inflammation of organ	− 2.1	< 0.001	38			
Energy production	Concentration of ATP	2.1	0.039	5			
Inflammatory response	Inflammatory response	2.3	0.006	19			
Cellular movement	Chemotaxis	2.6	0.020	13			
	Chemotaxis of leukocytes	2.6	0.003	12			
	Chemotaxis of myeloid cells	2.6	0.005	10			
	Chemotaxis of neutrophils	2.2	0.022	6			
	Chemotaxis of phagocytes	2.6	0.006	10			
	Migration of cells	2.9	< 0.001	71	− 2.6	< 0.001	56
	Leukocyte migration	2.3	< 0.001	54			
	Cell movement	3.1	< 0.001	79	− 3.0	< 0.001	62
	Cell movement of leukocytes	2.4	0.006	19			
	Cell movement of myeloid cells	2.5	0.007	15			
Vascular system development and function	Angiogenesis	3.3	0.024	19			
	Development of vasculature	3.3	0.036	20			
Infectious diseases	Viral Infection	3.6	< 0.001	37	− 2.8	0.002	24
	Replication of virus	3.2	0.003	15	− 2.6	0.010	11
	Replication of RNA virus	2.9	0.007	13	− 2.2	0.028	9
	Infection by RNA virus				-2.6	0.028	13

Cancer-related terms are excluded from the table

*Vpre*  preoperative visit, *Vpost* postoperative visit 1.5 h after surgery, *V1m* follow-up visit 1 month after surgery

### Relationship between clinical variables and the proteomics in myopic eyes

**Fig. 5:**
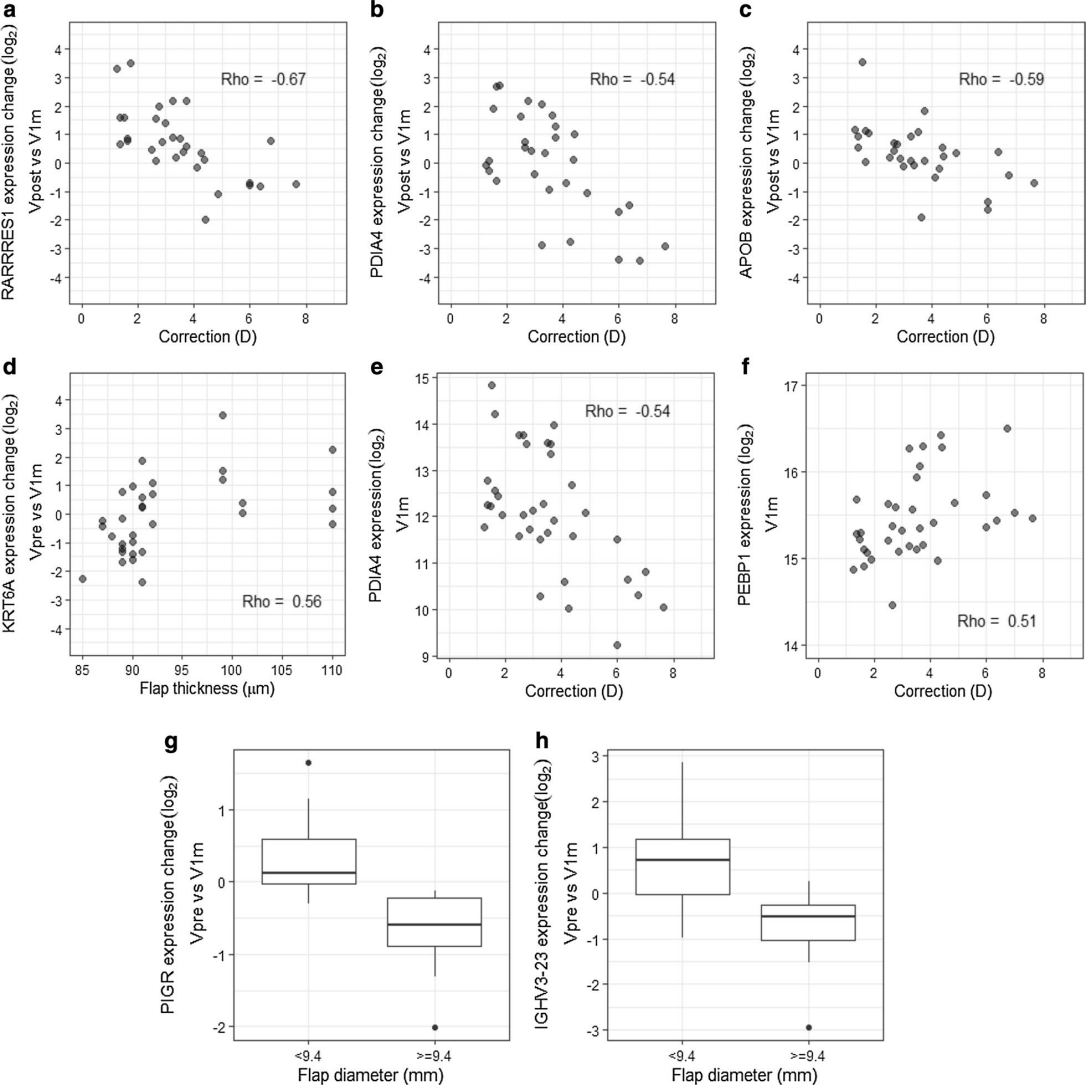
The amount of correction, flap thickness and flap diameter affect the protein expression levels. **a**–**c** Retinoic acid receptor responder protein 1 (RARRES1), protein disulfide-isomerase A4 (PDIA4), and apolipoprotein B-100 (APOB) expression level changes between Vpost and V1m time points correlated negatively with the amount of correction. **d** Flap thickness correlated positively with type II cytoskeletal 6A keratin (KRT6A) expression level changes between Vpre and V1m. **e**, **f** V1m expression levels of PDIA4 had a negative correlation and phosphatidylethanolamine-binding protein 1 (PEBP1) a positive correlation against the correction amount. **g**, **h** Flap diameter of 9.4 mm or over was associated with a decrease in expression levels of polymeric immunoglobulin receptor (PIGR) and immunoglobulin heavy variable 3–23 (IGHV3-23) 1 month after the surgery

Clinical variables, which are known to be connected to post-LASIK dry eye, i.e. sex as well as some surgical parameters such as the amount of correction, flap thickness and flap diameter were also evaluated in connection to the proteomics data. As seen from Fig. 5, several proteins correlated with the amount of correction and flap thickness. More specifically, retinoic acid receptor responder protein 1 (RARRES1), protein disulfide-isomerase A4 (PDIA4) and apolipoprotein B-100 (APOB) expression levels were noted to be more decreased 1 month after surgery for those patients, who had a greater attempted correction (Fig. 5a–c). Flap thickness also appeared to influence type II cytoskeletal 6A keratin (KRT6A) expression level changes 1 month after surgery in comparison to preoperative values (Fig. 5d).

In addition to examining the correlations between clinical variable and protein expression level changes, we also examined the correlations of clinical variables and protein expression levels in individual time points. According to our results, V1m expression levels of PDIA4 and phosphatidylethanolamine-binding protein 1 (PEBP1) were correlating with the amount of correction (Fig. 5e, f). Further results of the correlation comparisons are listed in Additional file 4.

Flap diameter was also examined in connection to protein expression levels, and a flap diameter of 9.4 mm or over appeared to influence the expression levels of polymeric immunoglobulin receptor (PIGR) and immunoglobulin heavy variable 3-23 (IGHV3-23). Both proteins were decreased significantly 1 month after surgery for patients with a larger flap diameter (Fig. 5g, h). Hinge length and sex were not affecting the protein expression levels according to our results.

## Discussion

It is known that corneal keratorefractive surgery affects both epithelial and stromal layers of the cornea and initiates a complex corneal wound healing cascade immediately after the surgery [9]. In the outermost epithelial layer, after an initial latent phase, high levels of adenosine triphosphate (ATP) are released and epithelial cells migrate to the edges of the wound in the early stages of wound healing [21, 22]. In the underlying corneal stroma, the wound healing is initiated by a rapid release of cytokines from the epithelium and lacrimal gland, causing stromal keratocyte apoptosis [10, 23, 24], followed by the remaining keratocytes’ proliferation to fibroblasts and myofibroblasts (reviewed by Wilson et al. [11]). In addition, migration of immune and inflammatory cells takes place within the first hours after corneal epithelial injury [25].

Our proteomics results indicated that tear fluid, from its part, enhances these biological processes early on, as we could identify a large collection of tear proteins, which had significantly altered expression levels 1.5 h after LASIK surgery. The pathway analyses further confirmed that these initial protein profile changes were connected to increased cell migration of immune cells, inflammatory response as well as concentration of ATP, which is required to fuel the increased energy consumption needed in all phases of the wound healing. In addition, the findings suggested increased activity in pathways connected to viral infection and angiogenesis. Despite the activation of angiogenic pathways in our results, the antiangiogenic signals are likely to balance these in order to maintain the avascularity, which is crucial for the normal function of cornea. Regarding the increased viral infection activity, there has been evidence that some, albeit a very small proportion of subjects, experience an activation of herpes simplex virus (HSV) keratitis after LASIK surgery [26, 27]. However, from the perspective of proteomics, this matter warrants more investigation.

The initial corneal wound healing effects, i.e. cell migration as well as keratocyte apoptosis and activation, peak within hours or days after surgery [25, 28], however, regeneration of nerve fibres, restoration of epithelial basement membrane and stromal remodelling may take from several months to years [29–31]. According to our tear proteomics results, approximately two-thirds of the proteins (104/158), which were identified to have altered expression levels 1.5 h after the surgery, had an expression level change of opposite direction 1 month after the surgery, while a third of the proteins did not display a similar return to normal. The proteins, which were observed to return to normal within the first month included various well-known tear fluid proteins. For example, ALB, ANXA1, ANXA2, ENO1, S100A4, S100A6, S100A9, actin, cytoplasmic 1 (ACTB), alpha-1-antitrypsin (SERPINA1), aldehyde dehydrogenase, dimeric NADP-preferring (ALDH3A1) and glutathione S-transferase P (GSTP1) were initially upregulated after the LASIK surgery, but expression levels had decreased back by the 1 month visit. According to previous studies, the expression levels of these proteins have been observed to be increased in aqueous or Sjögren syndrome dry eye, or both [32–36], thus being possible tear fluid indicators of ocular surface stress and possible inflammation. Our results, showing these proteins return to normal, therefore indicate that the acute state in the tear film has passed within the first postoperative month.

In addition to the inflammation-associated proteins, several keratins (KRT1, KRT4, KRT5, KRT7, KRT9, KRT13, KRT14, KRT19, KRT77), which are known to be vital structural proteins of epithelial cells, were increased initially. The observed expression levels indicate that the keratins are released to the tear fluid as a result of the wounding, which is expected. The expression levels of keratins were then reduced back to preoperative levels during the first postoperative month according to our results.

The results also included a collection of proteins, which initially had decreased protein expression levels 1.5 h after surgery, and which were then increased to preoperative levels within the first postoperative month. This protein group consisted almost entirely of immunoglobulin subunits, or associated proteins, and interestingly, previous studies have linked increased immunoglobulin expression levels with various ocular surface diseases and prolonged contact lens wear [37, 38]. The reasons for decreased levels of immunoglobulins in acute stress in our results remain unclear. One hypothesis could be that the tear fluid protein profiles 1.5 h after surgery could also be connected to surgery-associated eye drops, therefore affecting proteins connected to immune response and their expression levels. Alternatively, in the initial stages of the wound healing, i.e., hours after the surgery, the immunoglobulins may be bound to the wounded tissue or their production could take longer, thus causing them not being observed in the tear fluid.

According to our results, a third of the proteins with initially altered protein expression levels did not return to preoperative levels within 1 month. However, it is worth noting that in many cases, the fold changes indicated a shift back to preoperative levels, but the p-values were above the threshold, likely to due to large variation. Therefore, we decided to focus on proteins, which had a strong altered expression level (FC > 1.5 or < 0.67 and *p* value < 0.05) in one comparison and no notable change in the other (FC < 1.25 and > 0.8 and *p* value > 0.05). This way, after careful peak checking three well-known ocular surface proteins were studied further. Two of the proteins were membrane mucins, MUC4 and MUC16, which had increased expression levels 1.5 h after surgery and were not displaying signs of recovery, i.e., decrease, at 1 month after surgery. Increased expression levels of mucins in tear fluid, often connected to epithelium barrier function, could indicate an increased shedding from the epithelium, possibly due to pro-inflammatory agents in the tear films, such as tumor necrosis factor (TNF) [39]. This would suggest that from the perspective of epithelium layer, some wound healing functions are still ongoing 1 month after surgery. In addition to mucins, we found that androgen-associated secretoglobin family 1D member 1 (SCGB1D1) had significantly decreased protein expression level changes 1.5 h after the surgery and no notable change from these levels 1 month after the surgery. Decreased expression levels of SCGB1D1, as well as other proteins belonging to the secretoglobin family, have previously been connected to Meibomian gland dysfunction and evaporative dry eye [40, 41]. Overall, it would appear, that a small number of tear fluid proteins does not return to preoperative levels within 1 month, and it could be of interest to study these proteins further in relation to the development of adverse effects and more efficacious post-LASIK treatment methods.

In addition to differences between time points, the correlations between various clinical variables and protein expression levels were also evaluated. According to our results, particularly the amount of correction had an effect on the expression levels and expression level changes of some proteins. RARRES1 and APOB, both connected to peroxisome proliferating activating receptor (PPAR) and fatty acid metabolism [42, 43], had greater decreases in expression levels 1 month after surgery in connection to larger amount of correction. Interestingly, since the different PPAR isoforms have been connected to inflammation and wound healing of cornea, their antagonists have been suggested as potential therapeutic targets in corneal wound healing [44–46]. The potential roles of RARRES1 and APOB to the corneal wound healing should therefore be further studied. In addition, PDIA4 correlated similarly with the amount of correction. PDIA4 is closely connected to platelet activation as well as fibrin formation and thrombosis [47], however, this protein’s connection to the healing process of avascular cornea, are yet unknown. According to these correlation results, a greater amount of correction is connected to a decrease of several proteins closely connected to wound healing and associated inflammation, possibly indicating differing wound healing response caused by the deeper stromal excimer laser ablation in greater corrections. This may reflect differences in density and function of keratocytes and nerve fibers in different stromal layers.

Flap parameters were also associated to the expression level changes of proteins. Flap diameter affected the expression level changes of PIGR and IGHV3-23, both molecules connected to immunoglobulins. More specifically, when the flap diameter was 9.4 mm or larger, there was a notable decrease in expression levels 1 month after surgery, while with smaller diameters, the expression levels did not change or increased. As discussed earlier, our results showed that 1.5 h after LASIK surgery, many immunoglobulins were initially decreased, followed by an increase back to normal within 1 month. This additional observation indicating that not all proteins return to preoperative levels for some subjects with a larger flap diameter, could suggest that larger flap diameter, which could be connected to wider corneal nerve damage, does have an effect on the ocular surface recovery.

The LASIK-induced effects in the tear fluid have been studied previously to some extent, mainly through various cytokine studies [12, 13, 15, 16], but only one previous publication has evaluated the LASIK’s effects on larger protein profile [14]. In the study by D’Souza et al., the focus was on comparison of two types of femtosecond lasers, Visumax and Intralase, at later time points (1 week and 3 months) and thus, the initial changes taking place in the tear film were not examined. The results obtained from the D’Souza’s study and our study are not fully comparable also due to different sample collection methods (Schirmer’s strip vs capillary) and mass spectrometry methodology (iTRAQ vs SWATH-MS), which can both affect the protein identification and thus, protein profile results [18, 48]. Therefore, to our knowledge, our study is the first discovery proteomics study focusing on the initial functional changes occurring in the tear film after a LASIK surgery.

In surgery, medication is a vital and unavoidable part of the operation and always prone to influence the biological processes taking place during and after the surgery. In this study, the perioperative levofloxacin, diclofenac, brimonidine tartrate, oxybuprocaine hydrochloride and postoperative dexamethasone and chloramphenicol are likely to have at least short-term effects on the biological functions of ocular surface as well as the tear fluid composition, thus potentially affecting the postoperative samples’ protein profiles. However, as the drugs are a vital and standardized part of the LASIK surgery and as the ocular surface during and after LASIK surgery is not comparable to a normal, healthy ocular surface, examination of the pharmaceutical treatments’ effects alone is not plausible. The pre-, peri- and postoperative topical drugs are therefore important and necessary factors when evaluating the tear protein profile changes and altered biological functions after LASIK.

Overall, our findings help us understand better the initial biological changes taking place in the ocular surface after LASIK surgery. In the future, both the biological functions and individual proteins identified in our study could benefit the development of methods used to follow the post-LASIK healing process and to identify subjects, who do not recover as expected. In addition, knowing the changes taking place in tear protein profiles after a successful LASIK operation, could help develop new treatment approaches to prevent or reduce LASIK-related adverse effects.

## Conclusions

The present study showed that an uneventful femtosecond LASIK refractive surgery induced significant migration and inflammation-associated changes in tear proteomic profile quickly after the operation, but the expression of majority of proteins recovered almost completely to the preoperative levels during the first month. A small subset of proteins did not display similar return to normal, demonstrating that some wound healing functions remain ongoing also in the tear fluid 1 month after the operation. We also identified several proteins, which correlated in myopic eyes with the amount of refractive correction, flap thickness and flap diameter, indicating that the surgical variables affect the long-term, individual wound healing responses. The individual proteins identified in our study are potential targets for the follow-up and modification of LASIK-induced biochemical processes.

## Supplementary information

**Additional file 1.** Number of peptides used in the quantitation of proteins.

**Additional file 2.** Proteins with significantly changed expression levels (adj. P-value < 0.05) between Vpre and Vpost time points.

**Additional file 3.** Proteins with significantly changed expression levels (adj. P-value < 0.05) between Vpost and V1m time points.

**Additional file 4.** Correlations between clinical variables and protein expression levels performed with Spearman's correlation. Listed are only proteins with Rho > 0.5 or < -0.5.

## Data Availability

The datasets used and analysed during the current study are available from the corresponding author on reasonable request.

## Abbreviations

ACTB: Actin, cytoplasmic 1
ALB: Albumin
ALDH3A1: Aldehyde dehydrogenase, dimeric NADP-preferring
ANXA1: Annexin 1
ANXA2: Annexin 2
APOB: Apolipoprotein B-100
ATP: Adenosine triphosphate
CDVA: Corrected distance visual acuity
DDA: Data dependent-acquisition
ECM: Extracellular matrix
ELISA: Enzyme-linked immunosorbent assay
ENO1: Alpha-enolase
FC: Fold change
FDR: False discovery rate
GSTP1: Glutathione S-transferase P
HSV: Herpes simplex virus
IAA: Iodoacetamide
ICC: Intraclass correlation
IGHV3-23: Immunoglobulin heavy variable 3-23
IOP: Intraocular pressure
IPA: Ingenuity Pathway Analysis
iTRAQ: Isobaric tags for relative and absolute quantitation
KRT6A: Type II cytoskeletal 6A keratin
LASIK: Laser in situ keratomileusis
PDIA4: Protein disulfide isomerase A4
PEBP1: Phosphatidylethanolamine-binding protein 1
PIGR: Polymeric immunoglobulin receptor
PPAR: Peroxisome proliferating activating receptor
RARRES1: Retinoic acid receptor responder protein 1
SCGB1D1: Secretoglobin family 1D member 1
SDS: Sodium dodecyl sulphate
SEQ: Spherical equivalent refraction
SERPINA1: Alpha-1-antitrypsin
SWATH-MS: Sequential window acquisition of all theoretical fragment ion spectra mass spectrometry
TCEP: Tris-(2-carboxyethyl)phosphine
TNF: Tumor necrosis factor
UDVA: Uncorrected distance visual acuity
YWHAB: 14-3-3 Protein beta/alpha
YWHAZ: 14-3-3 Protein zeta/delta
